# Gut microbes differ in postmenopausal women responding to prunes to maintain hip bone mineral density

**DOI:** 10.3389/fnut.2024.1389638

**Published:** 2024-04-18

**Authors:** Abigayle M. R. Simpson, Mary Jane De Souza, Janhavi Damani, Connie J. Rogers, Nancy I. Williams, Connie M. Weaver, Mario G. Ferruzzi, Cindy H. Nakatsu

**Affiliations:** ^1^Department of Agronomy, Purdue University, West Lafayette, IN, United States; ^2^Department of Kinesiology, The Pennsylvania State University, College Park, PA, United States; ^3^Intercollege Graduate Degree Program in Integrative and Biomedical Physiology, Huck Institutes of the Life Sciences, The Pennsylvania State University, College Park, PA, United States; ^4^Department of Nutritional Sciences, The Pennsylvania State University, College Park, PA, United States; ^5^School of Exercise and Nutritional Sciences, San Diego State University, San Diego, CA, United States; ^6^Arkansas Children’s Nutrition Center, University of Arkansas for Medical Sciences, Little Rock, AR, United States

**Keywords:** prune, phenolics, bone mineral density, osteoporosis, osteopenia, menopause, gut microbiome, responder

## Abstract

Foods high in phenolics such as prunes have been shown to exert protective effects on bone mineral density (BMD), but only certain individuals experience these benefits. This *post-hoc* analysis of a 12-month randomized controlled trial aimed to identify the relationship among the gut microbiome, immune responses, and bone protective effects of prunes on postmenopausal women. Subjects who consumed 50–100 g prunes daily were divided into responders (*n* = 20) and non-responders (*n* = 32) based on percent change in total hip bone mineral density (BMD, ≥1% or ≤−1% change, respectively). DXA scans were used to determine body composition and BMD. Immune markers were measured using immunoassays and flow cytometry. Targeted phenolic metabolites were analyzed using ultra performance liquid chromatography-tandem mass spectrometry. The fecal microbiota was characterized through 16S rRNA gene PCR amplicon sequencing. After 12 months of prune consumption, anti-inflammatory markers showed responders had significantly lower levels of IL-1β and TNF-α. QIIME2 sequence analysis showed that microbiomes of responders and non-responders differed in alpha (Shannon and Faith PD, Kruskal-Wallis *p* < 0.05) and beta diversity (unweighted Unifrac, PERMANOVA *p* < 0.04) metrics both before and after prune treatment. Furthermore, responders had a higher abundance of bacterial families Oscillospiraceae and Lachnospiraceae (ANCOM-BC *p* < 0.05). These findings provide evidence that postmenopausal women with initial low BMD can benefit from prunes if they host certain gut microbes. These insights can guide precision nutrition strategies to improve BMD tailored to diet and microbiome composition.

## 1 Introduction

Menopause is associated with a rapid decline in bone mineral density (BMD) and deterioration in bone quality, thus compromising bone strength and predisposing postmenopausal women to increased risk for osteoporosis and fragility fractures. Dietary interventions are aimed at prevention of bone loss or as co-therapies to osteoporosis treatment. Calcium and vitamin D supplementation is considered a standard nutritional therapy for managing fracture risk (1, 2). There is increasing interest in leveraging select foods and bioactive food components for ameliorating bone loss and reducing fracture risk. Accumulating evidence suggests that foods rich in phenolic compounds, such as prunes, blueberries, and soybeans may be effective in protecting against postmenopausal bone loss (3). The exact mechanisms underlying the osteoprotective effects of phenolic-rich foods and supplements are largely unknown but are thought to be partly attributed to the ability of host and/or microbial phenolic metabolites to alter endogenous antioxidant capacity, to exert anti-inflammatory effects, or to provide prebiotic-like modulation of the gut microbiome (4–7).

The gut microbiome is a likely modulator of the effects of diet on BMD. In rodent studies, estrogen depletion was associated with loss in bone volume fraction in conventionally raised mice but not in germ-free mice (8), suggesting that the gut microbiome exerts a causal effect on BMD (9). The ability of dietary blueberries to counter estrogen deficiency-induced bone loss in female mice was associated with enhanced endogenous antioxidant response and increased gut microbiome diversity (6). Furthermore, observational studies in postmenopausal women have likewise noted that low BMD is associated with alterations in the gut microbiome (10–12). The gut microbiome may impact bone health through nutrient uptake, metabolite bioavailability, and immune regulation (13). Therefore, several factors such as diet, gut microbiome, and microbiota-derived anti-inflammatory phenol metabolites may collectively play a role in postmenopausal bone loss prevention. An understanding of these relationships is necessary for developing alternative non-pharmacological treatments for bone loss. However, limited work has been done in humans to elucidate the mechanistic associations among diet, gut microbiome, inflammatory markers, phenol metabolism, and bone health.

An emerging area of interest in precision nutrition is determining which factors predict an individual’s response to diet. In clinical studies with dietary interventions, participants are often observed to differentially respond to treatment, with subjects exhibiting improved changes in health outcomes termed as “responders” and those exhibiting no change or an unfavorable change are identified as “non-responders.” While clinical tools such as the Fracture Risk Assessment Tool (FRAX) are used to manage bone health and assess fracture risk after accounting for personal demographic risk factors (14), much less is known about which host characteristics and/or mechanistic factors predict bone response to dietary interventions. Several studies have identified responders to pharmacological treatment for osteoporosis. In a retrospective cohort study of 82 Mexican postmenopausal women with osteoporosis, investigators identified an osteogenomic profile unique to responders after 12 months of anti-resorptive bisphosphonate alendronate treatment (15). Furthermore, in a larger cohort study of 145 French postmenopausal women with osteoporosis, after 18 months of anabolic teriparatide treatment, non-responders had low levels of bone remodeling biomarkers, as assessed by C-terminal fragment of type 1 collagen (CTX) (16). Studies evaluating the role of the gut microbiome in predicting response to treatment often focus on few pre-existing or enriched bacteria with unique functions (17–21). However, multiple members of microbiomes may contribute to treatment outcomes. This requires studies that investigate entire microbial communities instead of select species.

In our parent randomized controlled trial (RCT), The Prune Study, we have previously reported that 12-month prune supplementation preserved total hip BMD in postmenopausal women and also exerted differential effects on phenolic metabolites, various inflammatory markers, and the gut microbiome (22, 23). However, our understanding of the relationship between microbiome composition and preservation of total hip BMD in response to prune supplementation in this trial is limited. Therefore, in the current investigation, we conducted a *post-hoc* analysis of secondary outcome data from the parent RCT, and specifically, we aimed to (1) identify the proportion of postmenopausal women who either responded or did not respond to the 12-month prune intervention for BMD preservation, (2) evaluate differences in the gut microbiome profiles between responders and non-responders at baseline and after 12 months of prune supplementation, and (3) determine which host factors or mechanistic factors, including baseline health, urinary phenolic metabolites, inflammatory markers, and gut microbiome, were associated with the 12-month change in total hip BMD from baseline. We hypothesized that differences in the microbiomes of responders versus non-responders contributed to the improved total hip BMD in a subset of participants from this prune study. To address these aims, postmenopausal women who consumed prunes daily for 12 months were divided into responder and non-responder groups based on percent change in hip BMD. Hip bone mineral density, the fecal microbiome, select urinary phenolic metabolites, and host inflammatory markers were analyzed at baseline and month 12.

## 2 Materials and methods

### 2.1 Study design

The Prune Study (Clinical Trials NCT02822378) was a parallel-arm, 12-month RCT conducted to evaluate the effect of a whole prune intervention on BMD, bone quality and estimated strength in postmenopausal women. The study was approved by the Pennsylvania State University (PSU) Institutional Review Board (IRB), and we have complied with all relevant ethical regulations. Informed consent was obtained during an in-person visit. A detailed description of the study design, inclusion/exclusion criteria, and methods (22); the primary outcome, bone health (24); and some secondary outcomes including the gut microbiome (23) have been published elsewhere. Briefly, eligible participants were postmenopausal women aged 55–75 years, not severely obese (BMI < 40 kg/m^2^), healthy (as determined by screening questionnaire and metabolic panel) and had BMD T-scores for DXA measures of the lumbar spine, total hip, and/or femoral neck between 0.0 and −3.0. Exclusion criteria included consumption of any phenolic-containing dietary supplement, a history of vertebral fracture or fragility fractures after age 50, or significant chronic disease. The subjects were randomly assigned to one of three treatment groups: a no-prune control group, 50 g prunes/day (4–6 prunes), and 100g prunes/day (10–12 prunes) for 12 months. Prunes were supplied by the California Prune Board. All participants were given supplements to meet the required daily intake of 1200 mg calcium and 800 IU vitamin D_3_ (Nature Made Pharmavite LLC, West Hills, CA). The nutritional composition of the prune interventions and run-in periods are described elsewhere (24).

#### 2.1.1 Body composition and bone mineral density

At baseline and week 52, body composition and total hip BMD were assessed using DXA. All participants underwent total body DXA scans on a Hologic QDR4500 system (Hologic, Bedford, MA, USA) performed by an International Society for Clinical Densitometry certified bone densitometry technologist. Laboratory precision for DXA measurement of total hip BMD was <0.8% coefficient of variation.

#### 2.1.2 Surveys

Demographic and medical histories were collected using in-house questionnaires. Three-day diet and 7-day exercise surveys were conducted at baseline and 12 months. The Nutritionist Pro™ Diet Analysis software (Axxya Systems, Redmond, WA, USA) was used to code and analyze nutrient data from diet logs.

#### 2.1.3 Selection of responders and non-responders

Previously, fecal microbiome analysis was conducted on 155 participants (58 Control, 58 50g, 39 100g) (23). The current investigation is a *post-hoc per-protocol* analysis conducted in a subset of participants only from the 50g and 100g prune groups who were ≥ 80% compliant to the prescribed prune dose and had gut microbiome data. Responders to prune supplementation were operationally defined as participants who exhibited at least 1.0% increase in total hip BMD from baseline (*n* = 20). This 1.0% definition was defined based on expected 12 month increase in hip BMD according to previous work on prunes and calcium and vitamin D interventions (25, 26). However, this is less than the 3–5% cutoff recommended by the American Association of Clinical Endocrinologists and that defined by studies of responders to drug trials (15, 16). This 1% cutoff allows for a larger sample size and accurate representation of responders to a dietary intervention. Non-responders exhibited at least 1.0% decrease in total hip BMD from baseline (*n* = 32). Some individuals in the no-prune control group gained total hip BMD on par with prune responders. To clearly characterize the prune response, and in particular draw distinctions between prune responders and individuals in the no-prune control group who gained BMD, we conducted parallel analyses in the no-prune control group (Supplementary Table 1). In the text, these individuals are labeled as BMD(+) (at least 1.0% increase in total hip BMD, *n* = 9) and BMD(−) (at least 1.0% decrease in total hip BMD, *n* = 28). A CONSORT diagram is available in Supplementary Figure 1.

### 2.2 Microbiome analysis

#### 2.2.1 DNA extraction and 16S rRNA gene illumina sequencing

DNA was extracted from homogenized fecal samples using the FastDNA SPIN Kit for Soil (MP Biomedicals) according to the manufacturer’s instructions. The V3-V4 region of the 16S rRNA gene was amplified by PCR with primers 343F/804R using a step-out protocol as previously described (22). PCR was conducted using the Q5 High-Fidelity Master Mix (New England Biolabs) and PCR products were purified using the Axy-Prep Mag PCR clean-up kit (Axygen^®^, Corning). Purified PCR products were quantified using the QuantiFluor dsDNA System (Promega) and a NanoDrop 3300 spectrofluorometer, pooled in equimolar concentrations, and sent to the Purdue Genomics Facility for 2x 250 paired end sequencing using an Illumina MiSeq instrument.

#### 2.2.2 Analysis of 16S rRNA gene amplicons

Sequences were analyzed using QIIME2 (27). After trimming and demultiplexing, DADA2 (28) was used to trim bases for quality, merge reads and identify amplicon sequence variants (ASVs). ASVs were assigned taxonomies with a taxonomy classifier trained on the SILVA database (version 138) (29). Sequences were filtered as previously described (23). For all diversity analyses, sequences were rarified to the same depth. Differences in alpha (measures diversity within a sample, metrics used: observed features, Pielou’s evenness, Faith’s phylogenetic diversity (30) and beta diversity (measures diversity among samples, metrics used: Jaccard, Bray-Curtis, unweighted and weighted Unifrac (31) were tested between responders and non-responders at baseline and month 12 in QIIME2. Beta diversity was visualized using Principal Coordinates Analysis (PCoA) plots using the R package phyloseq (32). Analysis of Compositions of Microbiomes with Bias Correction (ANCOM-BC) (33) was used to find differences in the log-transformed counts of taxa between treatment groups.

### 2.3 Host factor analyses

#### 2.3.1 Blood biochemistry

Fasted blood draws were collected as described previously (22). Metabolic and lipid panels were conducted at a commercial laboratory (Quest Diagnostics, Pittsburgh, PA, USA) from frozen plasma and serum aliquots.

#### 2.3.2 Excreted phenolic analysis using UPLC-MS/MS

At baseline and month 12, a 48-h pooled urine sample was analyzed to determine total phenolics and targeted phenolic metabolites using an ultra performance liquid chromatography-tandem mass spectrometer (UPLC-MS/MS) as previously described (22). Briefly, total urinary phenolics were determined from a 48-h pooled urine sample after solid phase extraction (SPE) extraction by the Folin-Ciocalteu microplate method (34) and corrected for creatinine content (colorimetric assay kit 500701, Cayman chemical, Ann Arbor, MI) (35). A targeted set of 21 prune derived phenolic metabolites were measured by UPLC-MS/MS using a Waters UPLC Acquity H Class system equipped with a Xevo TQD Mass Spectrometric detector.

#### 2.3.3 Immune marker assessment

Inflammatory markers were measured at baseline and month 12 as previously described (22, 36). Serum C-reactive protein (CRP) was measured using an Immulite (Siemens Healthcare, Munich, Germany) high-sensitivity CRP kit as per manufacturer’s instructions. Peripheral blood mononuclear cells (PBMCs) were isolated as previously described (22, 37). Cytokines and chemokines (IL-1β, IL-6, IL-8, TNF-α, and MCP-1) in plasma and supernatants harvested from lipopolysaccharide (LPS)-stimulated PBMCs were measured using the V-PLEX Proinflammatory Panel 1 Human Kit and V-PLEX Human MCP-1 kit (Meso Scale Diagnostics, LLC, Rockville, MD) as per manufacturer’s instructions. Plasma 8-isoprostane and serum total antioxidant capacity (TAC) concentrations were measured using Cayman’s 8-isoprostane kit and antioxidant assay kit, respectively (Cayman Chemicals) as per manufacturer’s instructions (intra- and inter-assay CVs were <10% for both assays). Each assay was performed in duplicate. The number and activation of circulating monocytes in PBMCs were quantified using flow cytometric analysis as previously described (38).

### 2.4 Statistical testing

Significant differences in alpha-diversity were tested using Kruskal-Wallis. Significant differences in beta diversity were determined using permutational multivariate analysis of variance (PERMANOVA) with 999 permutations. Permutational analysis of multivariate dispersions (PERMDISP) was used to determine if significant differences in beta diversity could be due to differences in the dispersion of groups instead of true differences in group means. *Post hoc* pairwise testing for all microbiome tests was FDR-corrected. For quantitative human health variables, normality was tested using Shapiro-Wilk’s method. Inflammatory markers were cleaned for outliers and normalized using log- or square root-transformations, as necessary. Unless otherwise noted, all significance testing among groups was done using ANOVA and subsequent pairwise testing by Tukey’s HSD. Linear correlations between the relative abundance of individual taxa and host variables of interest, namely total hip BMD, and IL-1β and TNF-α secretions from LPS-stimulated PBMCs, were determined using Spearman’s rank correlation coefficient in R with the package corrplot and FDR-corrected. For all analyses, p- or q-values less than 0.05 were considered significant, and those less than 0.10 are reported as trends.

## 3 Results

### 3.1 Characteristics of responders

#### 3.1.1 Differences in BMD

**FIGURE 1:**
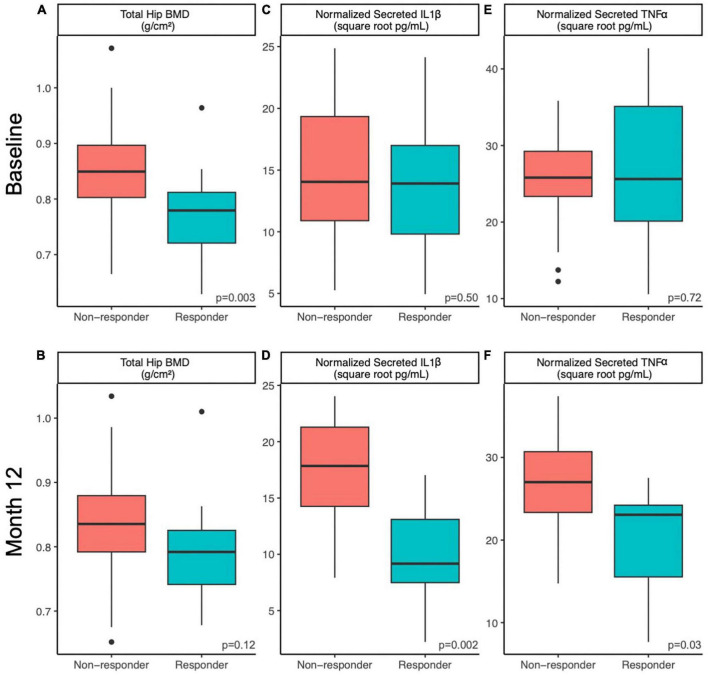
Host factors differ between responders and non-responders at baseline and 12 months. Differences in host factors **(A,B)** total hip BMD, **(C,D)** normalized PMBC-secreted IL-1β and **(E,F)** normalized PMBC-secreted TNFα between responders and non-responding postmenopausal women at baseline **(A,C,E)** and after 12 months **(B,D,F)** of prune consumption. Boxplots represent the medians and upper and lower quartile values, and whiskers extend to the minimum and maximum values, excluding outliers. Dots represent outliers. Responders (*n* = 20) and non-responders (*n* = 32). Student’s *t*-test values presented.

The baseline characteristics of 143 compliant participants who completed the 12-month RCT and had data for gut microbiome outcomes have been published elsewhere (23, 24). Of these 143 completers, 91 participants belonged to either of the prune interventions [50 g/day (*n* = 54) and 100 g/day (*n* = 37)]. Among this pooled prune group, we identified 20 responders [50 g/day (*n* = 13) and 100 g/day (*n* = 7)] and 32 non-responders [50 g/day (*n* = 20) and 100 g/day (*n* = 12)]. Baseline demographics such as age, BMI, dietary fiber intake, and prune consumption compliance did not significantly differ between responders and non-responders (Student’s t, *p* > 0.1, Table 1). At baseline, total hip BMD was lower among responders (mean ± standard deviation, 0.77 ± 0.07 g/cm^2^) than non-responders (0.84 ± 0.09 g/cm^2^, Student’s t, *p* = 0.003), but this difference was non-significant at month 12 of prune consumption (responders: 0.79 ± 0.07 g/cm^2^, non-responders: 0.83 ± 0.08 g/cm^2^, Student’s t, *p* = 0.12; Figures 1A, B). The change in total hip BMD differed between responders (+2.7 ± 2.5%) and non-responders (−1.9 ± 0.7%, Student’s t, *p* = 7.9E−8). This indicates that while BMD of responders increased (Student’s paired t, *p* = 3.6E−5), they also started at a lower BMD and did not have a final BMD higher than non-responders. A detailed visualization of within-individual changes in total hip BMD is shown in Supplementary Figure 2.

**TABLE 1:** Subject demographics of responders and non-responders.

	Non-responders	Responders	*p*
N	32	20	
Age (years)	62.5 ± 4.6	62.4 ± 5.6	0.90
BMI (kg/m^2^)	26.6 ± 4.4	24.9 ± 3.7	0.16
Dietary fiber intake (g/day)	21.9 ± 9.9	19.7 ± 8.1	0.41
Compliance (% of assigned prunes consumed)	95.9% ± 4.0%	95.4% ± 4.7%	0.69
**Treatment group**			
50 g prune group	20 (62.5%)	13 (65%)	
100 g prune group	12 (37.5%)	7 (35%)	

Metrics listed include, N (number of subjects), age, BMI (baseline body mass index), Dietary fiber intake (amount at baseline), Compliance (% of assigned prune treatment consumed), and Treatment group (amount of prune dose assigned). Mean ± standard deviations are presented. Significance values (not multiple-test corrected) for *t*-tests are presented.

#### 3.1.2 Differences in inflammatory markers and phenolic metabolites

Responders differed from non-responders on a number of different metrics. At week 52, IL-1β secretion from LPS-stimulated PBMCs was lower in responders (10 ± 5 square root pg/mL) compared to non-responders (17 ± 5 square root pg/mL, Student’s t, *p* = 0.002; Figures 1C, D). Likewise, TNF-α secretion from LPS-stimulated PBMCs at week 52 was lower in responders (20 ± 7 square root pg/mL) compared to non-responders (27 ± 6 square root pg/mL, Student’s t, *p* = 0.027, Figures 1E, F). The 12-month percent change in serum vitamin D levels from baseline was higher in non-responders (31 ± 30%) than responders (14 ± 25%, Student’s t *p* = 0.03). No other tested metrics were significantly different at baseline or week 52. It is worth noting that at baseline, responders tended to excrete less 4-hydroxybenzoic acid (Student’s t, *p* = 0.08), despite tending to consume more phenolic-containing foods as measured by a phenolic FFQ score (Student’s t, *p* = 0.07). However, after 12 months, responders consumed similar amounts of dietary phenols and excreted similar amounts of 4-hydroxybenzoic acid as non-responders (Student’s t, *p* > 0.25). No other excreted phenolic differed significantly between responders and non-responders.

### 3.2 Microbiome analysis

#### 3.2.1 Alpha diversity of prune-responder and non-responders differ

**FIGURE 2:**
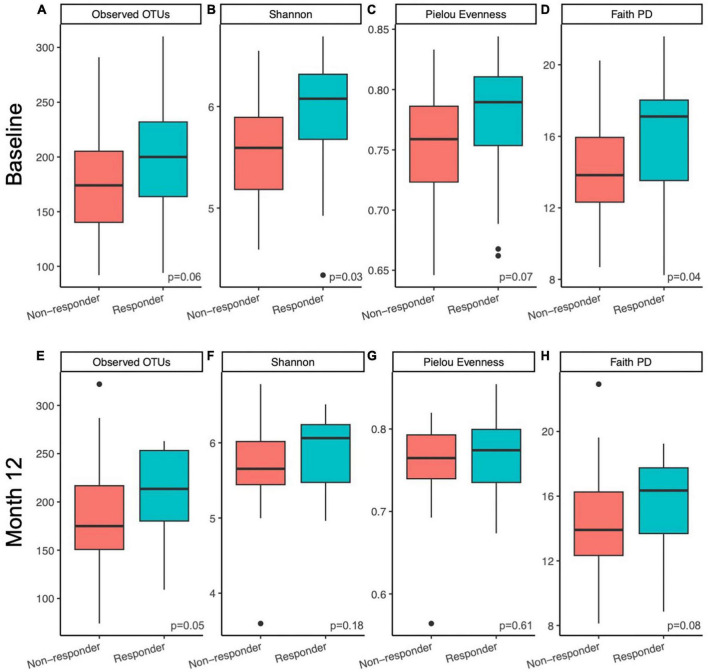
Microbiome alpha diversity was higher in responders compared to non-responders. Differences in within sample (alpha) diversity metrics **(A,E)** observed features, **(B,F)** Shannon, **(C,G)** Pielou Evenness, and **(D,H)** Faith PD between responders and non-responding postmenopausal women at baseline **(A–D)** and after 12 months **(E–H)** of prune consumption. Boxplots represent the medians and upper and lower quartile values, and whiskers extend to the minimum and maximum values, excluding outliers, represented by dots. Responders (*n* = 20) and non-responders (*n* = 32). Kruskal-Wallis significance *p*-values presented.

Alpha diversity of the gut microbial communities of responders and non-responders was compared at baseline and month 12. Responders had higher or trended higher in all alpha diversity metrics tested at baseline (observed features, Kruskal-Wallis *p* = 0.06; Shannon, Kruskal-Wallis *p* = 0.03; Pielou evenness, Kruskal-Wallis *p* = 0.07; Faith PD, Kruskal-Wallis *p* = 0.04; Figures 2A–D). At month 12, this difference was only maintained in observed features (measure of richness, Kruskal-Wallis *p* = 0.05; Figure 2E) and Faith PD (measure of phylogenetic diversity, Kruskal-Wallis *p* = 0.08; Figure 2H), but not Shannon (measure of richness and evenness, Kruskal-Wallis *p* = 0.18; Figure 2F) or Pielou evenness (measure of evenness, Kruskal-Wallis *p* = 0.61; Figure 2G). At month 12, responders had higher richness and more phylogenetically unique taxa compared to non-responders. Pairwise tests were conducted to determine whether responders had larger magnitudes of changes in alpha diversity than non-responders over the course of the prune intervention, but no significant differences were found (Kruskal-Wallis *p* > 0.44).

#### 3.2.2 Beta diversity shows microbiome differ among prune-responders and non-responders

**FIGURE 3:**
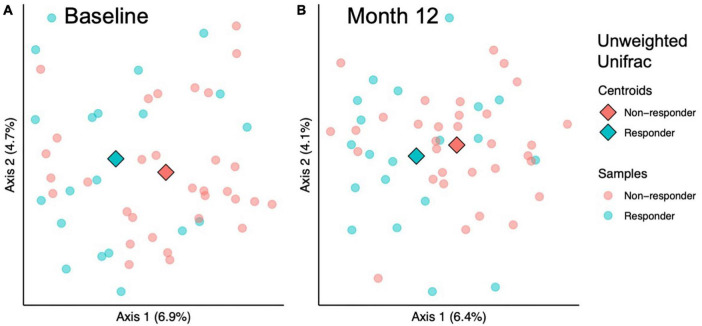
Beta diversity analysis indicates microbial community structure differs between responders and responders at baseline and 12 months. Among sample (beta) diversity of responders (*n* = 20) and non-responders (*n* = 32) at **(A)** baseline and **(B)** after 12 months of prune consumption. Principal coordinate plots of the beta diversity measured by Unweighted Unifrac. Each dot represents one microbiome, color coded by responder status. The centroids of each treatment group are represented by diamonds. PERMANOVA and PERMDISP *p*-values presented.

To investigate whether the gut microbial community structure of responders and non-responders differed significantly, beta diversity was compared at baseline and month 12 of the prune intervention. At baseline, responders differed from non-responders using Jaccard (considers taxa presence/absence; PERMANOVA *p* = 0.03) and unweighted Unifrac metrics (considers taxa presence/absence and phylogenetic diversity; PERMANOVA *p* = 0.04; Figure 3A). However, no differences were found using Bray-Curtis (considers taxa abundance; PERMANOVA *p* = 0.20) or Weighted Unifrac (considers taxa abundance and phylogenetic diversity; PERMANOVA *p* = 0.32). To verify that significant differences by PERMANOVA are due to true differences between groups and not due to differences in dispersion, PERMDISP was conducted, and found to be non-significant for all measures (*p* > 0.28). After the 12-month prune intervention, a similar pattern was found: metrics that only considered taxon presence or absence found significant differences (Jaccard, PERMANOVA *p* = 0.004; unweighted Unifrac, PERMANOVA *p* = 0.01, Figure 3B), while metrics that consider abundance did not (Bray-Curtis, PERMANOVA *p* = 0.55; weighted Unifrac, PERMANOVA *p* = 0.52). While PERMDISP trended significant for the Jaccard metric (*p* = 0.099), PCoA visualization reveals the samples clustered separately, indicating there are likely true differences in the groups. No other PERMDISP test was significant (*p* > 0.15). Taken together, these results indicate that differences in microbial community structures between responders and non-responders both at baseline and month 12 are likely due to taxa unique to either condition and not due to differences in relative abundances. Pairwise tests were conducted to determine whether responders experienced larger changes in community structure over the course of the prune intervention, but no significant differences were found (Kruskal-Wallis *p* > 0.59).

#### 3.2.3 ANCOM-BC identifies differentially abundant taxa in microbiomes of prune-responders and non-responders

**FIGURE 4:**
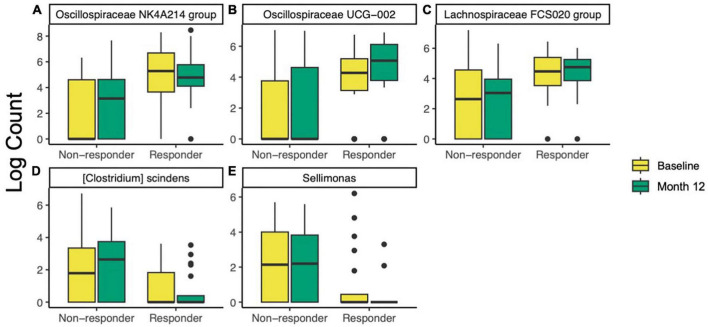
Differentially abundant taxa identified by ANCOM-BC in responders (*n* = 20) compared non-responders (*n* = 32) at baseline and after 12 months of prune consumption. **(A)** Oscillospiraceae NK4A214 (baseline *p* = 9.7E–5, 12 month *p* = 0.001), **(B)** Oscillospiraceae UCG-002 (12 month *p* = 0.021), **(C)** Lachnospiraceae FCS020 (12 month *p* = 0.002), **(D)** *{Clostridium} scindens* (*p* = 0.002), and **(E)** *Sellimonas* (*p* = 5.6E–4). Boxplots represent the medians and upper and lower quartile values, and whiskers extend to the minimum and maximum values, excluding outliers. Full list of differentially abundant taxa listed in Supplementary Table 2.

To further investigate differences in taxa between responders and non-responders, ANCOM-BC was used to test differences between responders and non-responders at baseline and 12 months. At baseline, two families, one genus, and two species were different or trended different between responders and non-responders (Supplementary Table 2). All five taxa were more abundant in responders. The most significant of these was Oscillospiraceae NK4A214 group (ANCOM-BC FDR-adjusted *p* = 9.7E−5, Figure 4A). After the 12-month intervention, four families, eleven genera, and seven species were different or trended different between responders and non-responders. Some taxa were consistently more abundant in responders at both time points, including Oscillospiraceae NK4A214 group (*p* < 0.002), Oscillospiraceae (*p* < 0.06), *Clostridium* UCG-014 (*p* < 0.06), and an uncultured organism in Oscillospiraceae UCG-002 (*p* < 0.10, Figure 4B). At month 12, Lachnospiraceae FCS020 group was more abundant in responders (*p* = 0.002, Figure 4C). Interestingly, some taxa were less abundant in responders at month 12, including *{Clostridium} scindens* (*p* = 0.002, Figure 4D), and *Sellimonas* (*p* = 5.6E−4, Figure 4E). To look for taxa that may be unique to either group that ANCOM-BC did not identify, an attempt was made to identify taxa that were detected in at least 75% of responders and less than 25% of non-responders (or vice versa). No taxa meeting these conditions were found at baseline or month 12. Further, ANCOM-BC did not identify any taxa whose abundance changed from baseline to month 12 within the responder or non-responder group.

### 3.3 Host and microbiome characteristics differentiating prune BMD response

#### 3.3.1 Associations between the gut microbiome and other subject measures

To evaluate potential relationships between BMD and specific taxa, linear correlations were conducted. To increase power and limit confounding effects due to responder status, all samples were included in this analysis, regardless of prune consumption or participant responder status (*n* = 310). Five taxa were significantly negatively correlated with hip BMD (Table 2), including Oscillospiraceae NK4A214 group (Spearman’s rho = −0.21, FDR-adjusted *p* = 0.03), an uncultured organism in Oscillospirales UCG-010 (Spearman’s rho = −0.20, FDR-adjusted *p* = 0.10), *Anaeroplasma* (Spearman’s rho = −0.19, FDR-adjusted *p* = 0.07), and Lachnospiraceae UCG-003 (Spearman’s rho = −0.21, FDR-adjusted *p* = 0.07). Likely due to low power, no taxa were significantly correlated with total hip BMD after FDR correction when only baseline samples (*n* = 155) or responders at month 12 (*n* = 20) were included. Further, linear correlations were conducted between all taxa and IL-1β and TNF-α secretions from LPS-stimulated PBMCs. One species, an uncultured organism in {*Eubacterium} coprostanoligenes* group, trended toward negative correlation with both cytokines (Spearman’s rho < −0.31; FDR-adjusted *p* < 0.08).

**TABLE 2:** Spearman’s correlations between log counts of taxa and total hip bone mineral density (BMD).

Order	Family	Genus	Species	Taxonomic Level	Spearman’s Rho	p	FDR-adjusted p
Oscillospirales	Oscillospiraceae	NK4A214_group	uncultured_rumen	Species	−0.23	3.2E−05	***0.02***
Oscillospirales	UCG-010	UCG-010	metagenome	Species	−0.20	4.0E−04	**0.10**
Oscillospirales	Oscillospiraceae	NK4A214_group	NA	Genus	−0.21	1.6E−04	***0.03***
Acholeplasmatales	Acholeplasmataceae	Anaeroplasma	NA	Genus	−0.19	6.8E−04	**0.07**
Lachnospirales	Lachnospiraceae	Lachnospiraceae_UCG-003	NA	Genus	−0.19	1.1E−03	**0.07**

Correlations were run at the species, genus, and family level (no family level correlations were significant). Only significant (*p* < 0.05 in bold italics) or trending (*p* < 0.1 in bold) correlations after multiple test corrections are presented.

#### 3.3.2 Characteristics of individuals who gained BMD in the no-prune control group

While prune consumption better preserved hip BMD compared to control, (39) it was noted that several individuals in the no-prune control group gained BMD on par with prune-consuming responders (Supplementary Figure 2). To further investigate this phenomenon, individuals in the no-prune control group who gained BMD (>1% increase in hip BMD, BMD(+), *n* = 9) or lost BMD (<−1% change in hip BMD, BMD(−), *n* = 28) were investigated to determine whether the no-prune control group displayed unique BMD-promoting characteristics compared to prune-consuming groups (Supplementary Table 1). BMD(−) and BMD(+) did not significantly differ in baseline BMD (Student’s t, *p* = 0.278). BMD(+) individuals had higher BMD at month 12 (Student’s t, *p* = 0.006) compared to BMD(−) individuals. Compared to BMD(−), BMD(+) individuals had higher BMI and LMI at baseline (Student’s t, *p* < 0.01), and had higher BMI, FMI, LMI, android fat, and visceral adipose tissue at month 12 (Student’s t, *p* < 0.05). Further, BMD(+) individuals had higher insulin and insulin resistance at baseline (Student’s t, *p* < 0.05), and higher blood glucose at both timepoints (Student’s t, *p* = 0.001). BMD(+) individuals consumed more of a number of micro- and macronutrients, including riboflavin and pantothenic acid (Student’s t, *p* < 0.04). BMD(+) individuals had lower serum Vitamin D at both time points (Student’s t, *p* < 0.03).

## 4 Discussion

In this *post-hoc* investigation of the Prune Study, we found that the gut microbiome and immunomodulatory signatures differed between responders and non-responders to prune supplementation. However, it is important to note that responders in this study started at a lower BMD than non-responders. Thus, it is difficult to determine whether differences in responders at baseline is associated with better ability to respond to prune supplementation, with lower BMD, or a combination of both. The primary difference in the gut microbiomes of responders and non-responders was the presence of phylogenetically diverse taxa, and this difference was preserved over the course of the 12 month prune treatment. Responders had higher alpha diversity, which may indicate more functional diversity and ability to metabolize prune components to beneficial metabolites. One model of responders and non-responders, specifically equol production from soy, is predicated on the presence of specific bacteria possessing the unique metabolic ability to produce equol (39, 40). The unique presence of bacteria able to metabolize prune components or otherwise modulate the host immune response may explain these results. Potential candidates include an uncultured organism in Oscillospiraceae UCG-002, which was, on average, undetected in non-responders but highly abundant in responders, or the *{Eubacterium} coprostanoligenes* group, which was negatively correlated with IL-1β and TNF-α secretions from PBMCs. Responders had lower levels of both cytokines after 12 months of prune supplementation. These cytokines are pro-inflammatory and associated with BMD loss (41, 42). While the role of *{Eubacterium} coprostanoligenes* in inflammatory signaling is unknown, these results suggest it may further decrease inflammatory cytokines and thus preserve BMD. However, further studies are needed.

*Moryella*, a member of the Lachnospiraceae, is likely one of the taxa to exert health benefits of prunes. Previous work with this dataset suggest *Moryella* is selected by prunes (23) and this genus was higher in responders at month 12. Lachnospiraceae are SCFA producers and immunomodulators (43, 44). *Moryella* has been isolated from abscesses (45, 46), but its major metabolic end products include SCFAs such as acetate and butyrate (45), which could indicate an anti-inflammatory role. More work is needed to determine the role of *Moryella* in the gut and in bone health. Broadly, this dataset supports existing literature that Lachnospiraceae is associated with BMD. We previously found Lachnospiraceae was of highest abundance in the 50g prune group (23), the group with the largest bone protection effect (24). Previous work has shown that Lachnospiraceae is positively associated with BMD and decreased fracture risk (47–49). However, it is worth noting that the taxa that were more abundant in responders were, for the most part, not increased by prune supplementation in this same data set (23). Similarly, organisms linearly correlated with hip BMD were not increased by prune supplementation, excluding Lachnospiraceae. This indicates that while prunes shift the gut microbiome, not all the shifts are directly associated with bone health.

Due to the baseline difference in hip BMD between the two groups, it is difficult to determine whether differences in the gut microbiome of responders at baseline is associated with better ability to respond to prune supplementation or with lower BMD. In fact, Oscillospiraceae NK4A214 group was both more abundant in responders and negatively correlated with BMD. For this reason, it is suggested that organisms such as Oscillospiraceae NK4A214 and Peptococcaceae, which were uniquely differentially abundant at baseline, may be associated with low BMD rather than ability to respond to treatment. Previous research showing a benefit of inulin-enriched inulin in postmenopausal women reported that responders have lower starting BMD than non-responders (50).

Prune supplementation appears to enhance hip BMD through distinct pathways compared to those not consuming prunes. In the no-prune control group, individuals who gained BMD had more fat and lean mass. While the connection between weight and BMD is still under study, BMD has been positively correlated with obesity (51), and it is thought that high BMI may protect BMD through increased bone loading and higher levels of bone-protective 17β-estradiol (52). However, increased BMI may lead to detrimental effects such as insulin resistance (53), and this effect was observed in the no-prune control. In contrast, prunes likely act through the gut microbiome and inflammatory pathways and do not depend on weight. In addition, all participants were supplemented with calcium and vitamin D, which may have its own responders and non-responders. Because it is likely that responders to calcium and vitamin D would have the same characteristics, regardless of prune consumption, we posit that the results described herein are likely due to prune response instead of calcium and vitamin D response. These results suggest that when combined with a vitamin D and calcium supplements, prunes may be a promising whole-food nutritional supplement to maintain BMD in postmenopausal women. In the context of precision nutrition, baseline characteristics of our subjects suggest that individuals with lower BMD, high fecal microbiota alpha diversity, and specific bacteria such as Oscillospiraceae UCG-002 are more likely to benefit by including prunes in their diet for bone health.

Intervention and mechanistic studies investigating the relationship between prune, microbes, and immune markers are necessary to test the associations presented here. Due to the associational nature of this analysis, we were unable to determine whether some effects present in responders at baseline were due to lower BMD or higher ability to respond to prunes. Our conclusions are limited to the effects of whole prunes, and future studies may benefit from investigating components of prune, such as fiber or phenolics. Further, we were unable to isolate the effects of phenolic compounds from the effects of the prune-altered gut microbiome on host immune status, as both are likely involved in modulating immune status, potentially in a cyclical manner. Future mechanistic research can address these questions. Further limitations of this study include the limited demographic diversity of the subjects. To reduce variability, this study looked at a narrow segment of postmenopausal women, and more studies are required to capture the full breadth of prune response mechanisms among diverse postmenopausal women. While excreted phenolics, as potent immunomodulatory compounds and likely microbial metabolites of prunes, were expected to differ between responders and non-responders, none of those tested differed significantly, indicating that more targeted analysis of additional metabolites of prune or other dietary phenolic sources may be necessary. This study collected valuable information regarding the taxonomic identities of microbes involved in prune response, but more detailed analyses such as metagenomics or metatranscriptomics would provide additional information regarding microbiome functional changes of prune responders.

## 5 Conclusion

We identified several microbiome and human health variables associated with responders to prune supplementation. These factors may be involved in the mechanisms underlying the bone-protective effects of prune supplementation in postmenopausal women. These results suggest responders have more diverse gut microbiota, including organisms in Oscillospiraceae UCG-002 and *{Eubacterium} coprostanoligenes*, which may exert anti-inflammatory effects by attenuating IL-1β and TNF-α secretion from PBMCs. Lowered inflammation supports bone deposition, increasing BMD. We identified several organisms and host characteristics correlated with BMD, which could open future avenues of research into modulating BMD through precision nutrition.

## Data availability statement

Raw nucleotide sequences used in this study have been deposited in the National Center for Biotechnology Information-Short Read Archive (NCBI-SRA) database under accession number PRJNA1041947. The metadata described in this manuscript are available upon reasonable request from MJDS who ran the clinical trial. The full dataset has not been made publicly available because it contains information that can compromise research subject privacy and consent.

## Ethics statement

The studies involving humans were approved by the Pennsylvania State University Institutional Review Board. The studies were conducted in accordance with the local legislation and institutional requirements. The participants provided their written informed consent to participate in this study.

## Author contributions

AS: Formal analysis, Writing – original draft, Writing – review and editing. MD: Conceptualization, Data curation, Funding acquisition, Investigation, Project administration, Writing – review and editing. JD: Writing – review and editing. CR: Conceptualization, Methodology, Writing – review and editing. NW: Conceptualization, Writing – review and editing. CW: Conceptualization, Writing – review and editing. MF: Conceptualization, Methodology, Writing – review and editing. CN: Conceptualization, Data curation, Formal analysis, Investigation, Methodology, Project administration, Supervision, Writing – original draft, Writing – review and editing.
