# Supplementary material for: Gut microbes differ in postmenopausal women responding to prunes to maintain hip bone mineral density
Source: Front Nutr. 2024 Apr 18;11:1389638. doi: 10.3389/fnut.2024.1389638 (PMC11067506; doi:10.3389/fnut.2024.1389638)
Supplement: Supplementary file 3 [file Data_Sheet_1.docx]

**Supplemental Information**

Gut microbes differ in postmenopausal women responding to prunes to maintain hip bone mineral density

Abigayle M. R. Simpson,^1^ Mary Jane De Souza,^2^ Janhavi Damani,^3^ Connie Rogers,^4^ Nancy I. Williams,^5^ Connie Weaver,^5^ Mario G. Ferruzzi,^6^ Sydney Chadwick-Corbin,^6^ Cindy H. Nakatsu^1*^

^1^ Dept. of Agronomy, Purdue University

^2^ Dept. of Kinesiology, Pennsylvania State University

^3^ Intercollege Graduate Degree Program in Integrative and Biomedical Physiology, Huck Institutes of the Life Sciences, The Pennsylvania State University

^4^ Dept. of Nutritional Sciences, Pennsylvania State University

^5^ School of Exercise and Nutritional Sciences, San Diego State University

^6^ Arkansas Children’s Nutrition Center, University of Arkansas for Medical Sciences

*Corresponding author

**Supplemental Figures**

**
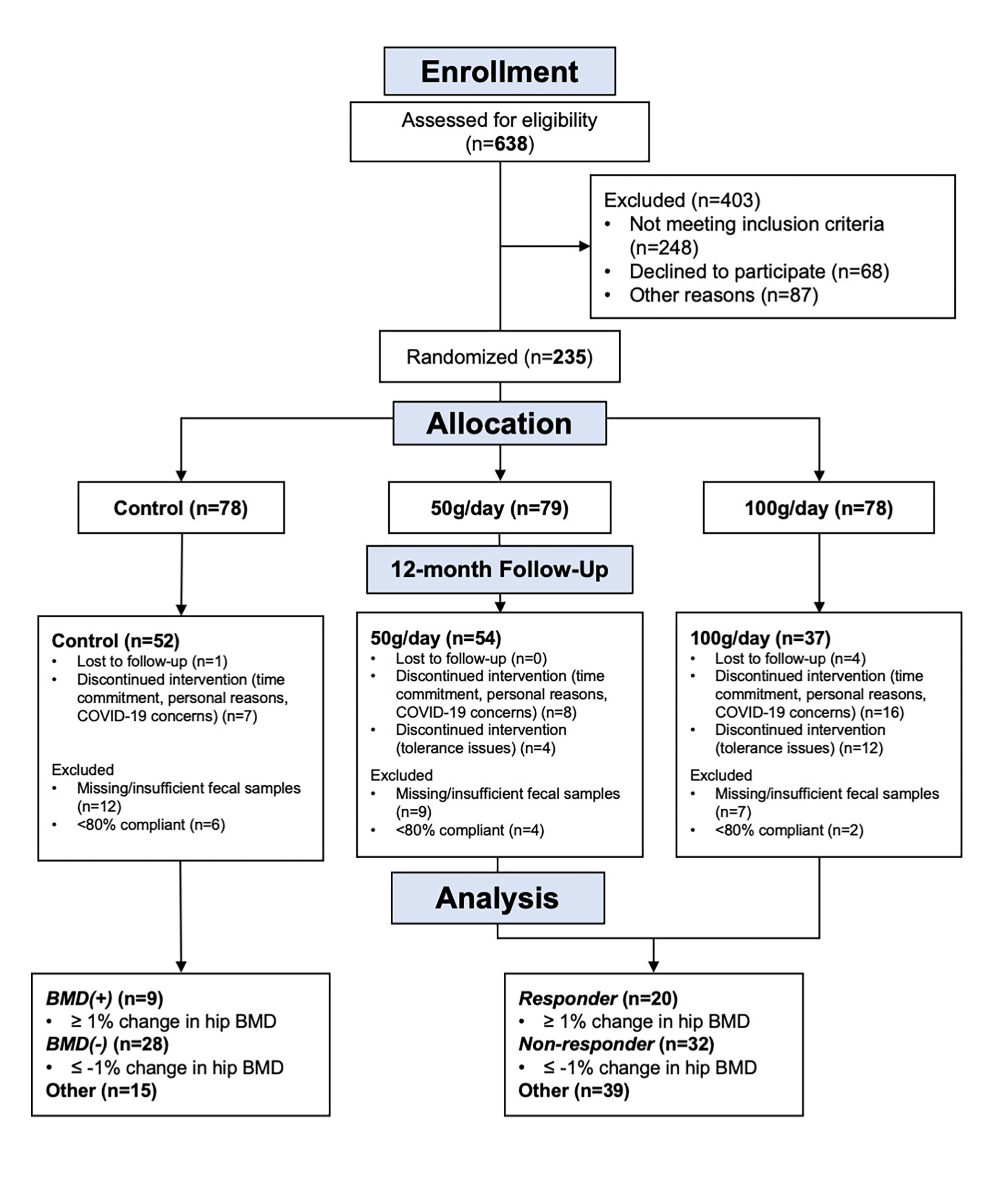
**

**Supplemental Fig. S1.** **CONSORT diagram**. This diagram illustrates the number of individuals allocated to each treatment, reasons for discontinuation or exclusion, and final analysis.


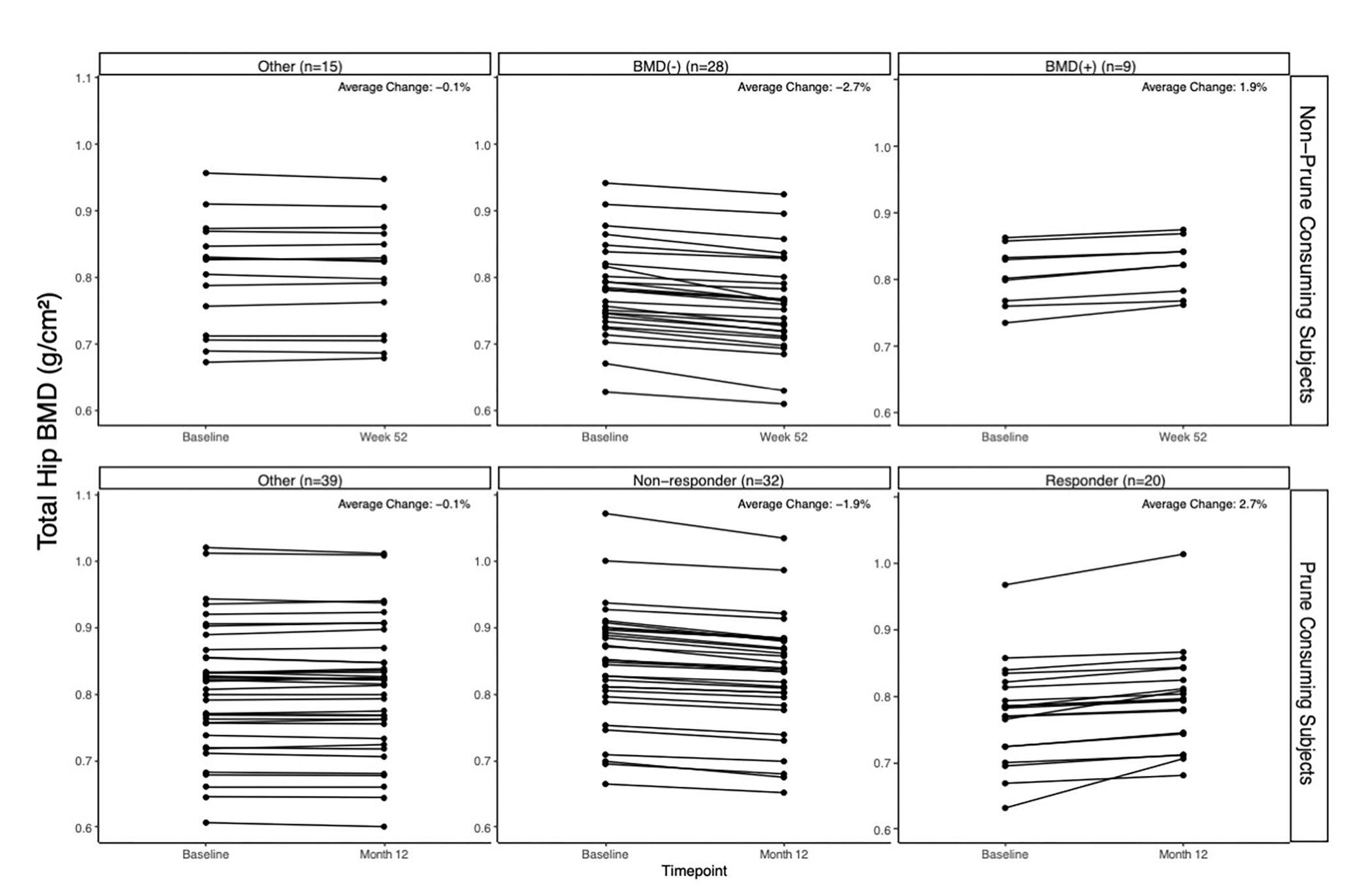


**Supplemental Fig. S2.** **Change in total hip bone mineral density (BMD) with and without prune intervention.** Change in total hip BMD from baseline to month 12 of those who gained BMD, lost BMD, and other subjects across prune-consuming and non-prune consuming groups. Each pair of dots and line represents one subject. Average change for each group is presented.

**Supplemental Tables**

**Supplemental Table S1:** Means and changes in host variables measured at baseline and 12 months.

See tables in Excel sheet attachments.

**Supplemental Table S2:** ANCOM-BC identifies significantly difference taxa in responders compared to non-responders at baseline and 12 months.

See attached excel table.

Data availability

Raw nucleotide sequences used in this study have been deposited in the National Center for Biotechnology Information-Short Read Archive (NCBI-SRA) database under accession number PRJNA1041947. The metadata described in this manuscript are available upon reasonable request from MJDS who ran the clinical trial. The full dataset has not been made publicly available because it contains information that can compromise research subject privacy and consent.
